# Interspecies Retinal Diversity and Optic Nerve Anatomy in Odontocetes

**DOI:** 10.3390/ani13213430

**Published:** 2023-11-06

**Authors:** Michiel W. E. De Boeck, Bruno Cozzi, Jean-Marie Graïc

**Affiliations:** 1 Independent Researcher, 9000 Ghent, Belgium; 2 Department of Comparative Biomedicine and Food Science (BCA), University of Padova, 35020 Legnaro, Italy; bruno.cozzi@unipd.it (B.C.); jeanmarie.graic@unipd.it (J.-M.G.)

**Keywords:** retinal ganglion cells, optic nerve, cetaceans, Odontoceti, retina, neurofilament 200, calretinin

## Abstract

**Simple Summary:**

This study aimed to contribute to the knowledge on the visual system in multiple toothed whales focusing on the neurological parts, the optic nerve and the retina. We hypothesised to find some of the characteristics typical for cetacean species while also seeing differences between different species and in comparison with close relatives: the bovine and common hippopotamus. Results from the optic nerve appeared to be very species specific, showing a general pattern of lower division into nerve bundles in the cetaceans and in the hippopotamus. The total area of the nerve that contained axons ranged from 75.11% in *Globicephala melas* to 96.18% in *Stenella coeruleoalba*. All the examined samples showed the typical layered structure of the retina with a mean thickness of 140.2 µm for *Grampus griseus* and 263.8 µm for bovine. The linear retinal ganglion cell density varied between 0 and 12 cells per mm in the odontocetes, while in bovine, it was considerably higher. The study presented here provided clear evidence of anatomical differences within toothed whale species and with reference species. However, multiple patterns observed here do not comply with what is known from the literature (i.e., low retinal thickness and absence of giant ganglion cells).

**Abstract:**

Throughout evolution, odontocete vision has had to readapt to the aquatic environment, which has had far-reaching effects on ocular anatomy and neurology. The most prominent features include the iris with an operculum, a well-developed choroid, the presence of giant ganglion cells in the retina, and the hemispherical shape of the thick eyecup. In the present study, the optic nerve and the retina were comparatively studied in Odontoceti (Cuvier’s beaked whale, common bottlenose dolphin, false killer whale, long-finned pilot whale, Risso’s dolphin, striped dolphin), the semi-aquatic common hippopotamus, and the fully terrestrial bovine. Cross-sections of the tissue were treated with histological and immunohistochemical techniques. Substantial differences were seen between the odontocetes and the reference species as well as within the cetaceans. The morphological structure of the optic nerve mainly appeared species specific, while the density of retinal ganglion cells was significantly higher in the terrestrial bovine than in the cetaceans. However, some typical characteristics of the cetacean retina were absent: the giant ganglion cells and the high retinal thickness. Immunohistochemical research showed varying degrees of neurofilament 200 expression in the retinal ganglion cells, while calretinin was only expressed in those of the common bottlenose dolphin and bovine.

## 1. Introduction

The ability to sense their environment has clear advantages for most animals, ranging from hearing an approaching predator to recognising the colourful pattern of a conspecific. Unlike other senses, the visual system delivers fast and accurate information about close-by as well as far-away cues. It ranges from simple photoreception to high acuity spatial vision, the latter restricted to certain arthropods, molluscs, and vertebrates [1,2]. Advanced sight required the transition from a flat receptor array to one or multiple cup-shaped ones, the membrane stacking of the photoreceptor cells, and the development of focusing optics, i.e., the lens [2].

In vertebrates, five classes of neural cells can be distinguished in the three-layered retina. The outer nuclear layer comprises the photoreceptors, which are hyperpolarised when activated by a photon. Next, the inner nuclear layer contains the interneurons: horizontal cells, bipolar cells, and amacrine cells; these enable within- and between-layer transmission of the signals. The retinal ganglion cells (RGCs) are located most inward and connect the retina with various brain regions via their axons, which form the optic nerve. Finally, these nuclear layers are alternated by plexiform layers where their dendrites and axons synapse with each other. For the interneurons, this happens in the outer plexiform layer (OPL) with the photoreceptors and the inner plexiform layer (IPL) with the ganglion cells [3,4]. 

The axons of the RGCs converge into the optic nerve, which is the second cranial nerve that leads to the forebrain. Targets in the brain include the primary visual cortex via the lateral geniculate nucleus in the thalamus, the superior colliculus, the Edinger–Westphal nucleus via the pretectum, and the suprachiasmatic nucleus [3]. The axons in these nerves are combined into fascicles, each enveloped by the perineurium; all bundles together are surrounded by the epineurium that condenses into the nerve sheath [5].

With the pioneering work of Cajal at the end of the 19th century, the existence of different morphological subtypes within these broad classes was brought to light [6]. When Lettvin et al. (1959) [7] subsequently found four groups of ganglion cells with different physiological responses to visual stimuli, the concept of specialised retinal circuitry finetuned to specific triggers was born. These filter out the essential visual information to lower the computational load on the brain and help prioritise the most critical signals [8]. Since its first conceptualisation, a rich research discipline into the circuitry of the retina in different species has blossomed, with a complete understanding of all types and how those interact with each other coming into sight for certain model organisms (e.g., in mouse [9]). 

Throughout evolution, only a few mammalian lineages have returned from the terrestrial environment to the aquatic world, including the cetaceans, pinnipeds, and sirenians. This has had severe effects on their sensory experience; the visual system in particular has had to adapt to the higher refractive index of water, the mechanical and chemical abrasiveness of the medium, and the high-pressure, low-temperature and low-light conditions [10]. The cetacean eye offers a unique opportunity to study how the visual system adapts and evolves under such intense changes. Even though in toothed whales, a substantial amount of their worldview is produced by their well-developed auditory system and echolocation abilities, vision still forms a meaningful sense, especially in shallow dwelling coastal groups. Orientation and navigation, prey detection and capture, communication of behavioural states, and recognition of characteristics of conspecifics still largely depend on their eyesight [11].

Within cetaceans, these conditions have led to some remarkable ocular features. The most prominent features include a thick sclera, highly developed ocular muscles, an extensive choroid layer, thickened cornea, and the hemispherical shape of the eyeball [10]. In addition, the ciliary muscles are absent or strongly reduced, leading to the notion that accommodation comes from lens movement by changing the pressure difference between the anterior and posterior chamber [10,12]. The thick and rigid sclera together with strong retractor bulbi are thought to be at the base of this process [13].

Thorough genetic and immunohistochemical research has revealed the loss of a short-wavelength-sensitive cone receptor in cetaceans through the inactivation of the SWS1 gene [14,15]. This leaves them with a high percentage of rod photoreceptors, which are functional at low light levels, and a sparse population of the long-wavelength-sensitive L-cones [16]. Both receptors have obtained a slight shift to shorter wavelengths in Delphinoidea (dolphins, porpoises, and beluga), which is indicative of adaptation to their blue-dominated visual environment [14]. A reflective tapetum lucidum is present on two-thirds of the whole ocular fundus and is of the fibrillar type, creating a significant amount of back radiation to the photoreceptors and so increasing the light sensitivity of the eye [10,17,18].

Since cetaceans experience substantial and rapid differences in illumination, the amount of light that reaches the retina must be adjusted accordingly. The iris has developed a characteristic “operculum” in its dorsal part, which can slide over the pupil. At very high light levels, only a rostral and temporal pinhole remains. These openings correspond with the two areas of higher neuronal density that have been found in many cetaceans [10,17,19,20,21,22]. The retina itself has the characteristic layered appearance of the mammalian retina, yet it has a thickness between 370 and 425 µm compared to 110 to 220 µm in the bovine [10]. A prominent difference with terrestrial mammals can be found in the ganglion cell layer: the RGCs are sparsely distributed and separated by wide intercellular spaces; however, some clustering into closely spaced cells can also be seen [10]. This results in a lower density of RGCs; Mass and Supin (1995) [19] report a peak density of 826 cells per mm^2^, while in the periphery, the density reached less than 150 cells per mm^2^ in the common bottlenose dolphin. In humans, RGC density reaches up to 37,800 cells per mm^2^ [23], while in the bovine, it reaches up to 6500 [24]. Additionally, these cells are remarkably large, ranging from 10 to 60 µm with a peak at 20 to 30 µm and giant cells reaching up to 75–80 µm [10]. Previous estimates for RGC density and size can be found for the species studied here in Table 1. 

Regarding the optic nerve, cetaceans show a remarkable reduction in fibre density coupled with the presence of very large axons, which is in line with the observations of their ganglion cells [10]. For example, Gao and Zhou (1992) [28] reported a mean fibre density of around 19,000 per mm^2^ in *Tursiops truncatus* with diameters ranging from 0.4 to 25.6 µm. In comparison, the human optic nerve has a mean axon density of 193,238 per mm^2^ [31] with diameters ranging from 0.57 to 1.63 µm [32]. Other reference estimates for the species covered in this study, if available, are shown in Table 1. Surrounding the optic nerve a rete mirabilis is present; this is a complex network of small arteries [17]. In cetaceans, it is involved in the maintenance of photoreceptor and ocular muscle functionality in cold ambient temperatures and possibly in conserving oxygen during long anaerobic dives [33]. 

The present study aims to contribute to the knowledge on the comparative anatomy of the cetacean eye through a two-fold approach. Firstly, the retina was studied through cross-sections stained with cresyl-violet and immunohistochemical procedures for calretinin and neurofilament 200, focussing on the retinal ganglion cells. Secondly, the optic nerve was looked at with a Weil stain for myelin and analysed with a focus on the nerve bundle division.

The species covered here include the following toothed whales: long-finned pilot whale (*Globicephala melas*), Risso’s dolphin (*Grampus griseus*), false killer whale (*Pseudorca crassidens*), striped dolphin (*Stenella coeruleoalba*), common bottlenose dolphin (*Tursiops truncatus*), and Cuvier’s beaked whale (*Ziphius cavirostris*). In addition, the bovine (*Bos taurus*) is used as a terrestrial reference species and the common hippopotamus (*Hippopotamus amphibius*) is used as a semi-aquatic one, all belonging to the Artiodactyla. For *G. griseus* and *Z. cavirostris*, this study represents the first of its kind.

We hypothesised the presence of apparent differences between the cetaceans and the reference species as described in the literature as well as some interspecies variety within the cetaceans, since vision is strongly influenced by the visual requirements of the animal as well as eye size and energy availability [34]. 

## 2. Materials and Methods

### 2.1. Tissue Collection and Storage

A total of nine samples from eight species were examined, details of which can be found in Table 2. Both bovine eyes came from the same animal; they were obtained from a commercial slaughterhouse and were treated according to the European Community Council directive (86/609/EEC) on animal welfare during the commercial slaughtering process while being constantly monitored under mandatory official veterinary medical care. Cetacean and hippopotamus (*Hippopotamus amphibius*) specimens were supplied by the Mediterranean Marine Mammal Tissue Bank (www.marinemammals.eu (accessed on 1 October 2023)) at the Department of Comparative Biomedicine and Food Science at the University of Padova. This organisation collects samples from cetacean stranding events along the Italian coast and those originating from marine theme parks or zoos for necropsy and post-mortem diagnosis.

The eyes and some additional intra-orbital tissues were extracted from the bodies and placed in a 4% neutral-buffered paraformaldehyde solution for initial fixation. The fixative was replaced at least one week before processing, and at the edge of the cornea an incision was made to aid penetration into the eye.

Since autolysis rapidly sets in after death and causes the degradation of biological tissues, quick fixation is essential. Therefore, estimated post-mortem intervals (PMIs) are given in Table 2; where this was not possible, the preservation status of the stranded animal is given. This score is given based on the internationally recognised system developed by Ijsseldijk et al. (2019) [35]. 

### 2.2. Sampling and Histological Processing

#### 2.2.1. Optic Nerve Cross-Sections

The optic nerve was separated from the surrounding tissue and washed in Tris-buffered saline solution (TBS, 50 mM Trizma base, 150 mM NaCl, pH 7.6; Sigma-Aldrich, Burlington, MA, USA) before going through automatic processing and paraffin embedding (Shandon Citadel 1000, Thermo Scientific, Waltham, MA, USA, #69,800,004). The resulting tissue blocks were then cut perpendicular to the direction of the nerve into 5 µm sections which were mounted on glass slides (Superfrost Plus, Menzel Gläser, Thermo Scientific, J1800AMNZ) and subsequently stained using the Weil method for myelin. In short, the samples were dewaxed and rehydrated before being put into the dye for 45 min at 56 °C, which consisted of an equal proportion mixture of aqueous iron alum (4% *w*/*v*) and fresh alcoholic haematoxylin (1%). The samples were then washed in distilled water, partially differentiated in another aqueous iron alum for two minutes, washed again, and fully differentiated in Weigert’s borax ferricyanide for one minute. After a final rinse with multiple changes of distilled water, the tissue was dehydrated through a graded alcohol scale, cleared in xylene, and coverslipped with Entellan medium.

#### 2.2.2. Retinal Cross-Sections

Each eye was prepared for processing by carefully removing the cornea, iris, and lens. Then, the vitreous body was removed as much as possible without disturbing the retina. The eye was hemisected along the dorsoventral axis, and two parallel strips were cut from one hemisphere, processed, and embedded in paraffin. The resulting blocks were cut perpendicular to the retinal surface into 8 µm sections and mounted on glass slides (Superfrost Plus, Menzel Gläser, Thermo Scientific, J1800AMNZ). Three consecutive sections spanning the full eyecup dorsoventrally and taken 1–2 mm from the optic nerve entrance were used for different protocols; the first section was subjected to a simple Nissl stain with cresyl-violet.

Briefly, sections were dewaxed in xylene and hydrated in a descending alcohol series before a rinse in distilled water and slight acidification in a 5% acetic acid solution. Then, the slides were placed in a 2% cresyl-violet solution for five minutes, rinsed in distilled water, and partially dehydrated before passing through a differentiation solution (0.1% acetic acid in 95% ethanol) for one minute. Hereafter, dehydration was completed in 100% alcohol, after which the slides were cleared in xylene and coverslipped in mounting medium.

### 2.3. Immunohistochemistry

The remaining two paraffin sections (8 µm) of the eyecup were subjected to immunohistochemistry. Positive immuno-reaction was achieved using two antibodies: monoclonal mouse anti-calretinin (CR, 1:500, Swant, Basel, Switzerland) and monoclonal mouse anti-neurofilament 200 (N200, 1:500, Sigma-Aldrich, Burlington, MA, USA) (see Table 3). Calretinin is part of the calcium-binding proteins, while neurofilament 200 forms the large subunit of the neurofilament fibre. Antigen retrieval was accomplished by microwave-aided heating to a temperature of 90 °C for 10 min in a 0.05 M Tris/HCl buffer solution of pH 9.0. After a rinse in TBS (3 × 5 min), a 1% hydrogen peroxide solution in TBS was applied for 10 min to block endogenous peroxidase activity. To ensure proper antibody binding, the samples were incubated with the solutions containing the primary antibodies in supermix (SuMi: 0.5% Triton X-100 and 0.25% gelatin in TBS) for one hour at room temperature and then overnight at 4 °C. Next, the sections were incubated in the biotinylated secondary anti-mouse antibodies (1:400, 5 µg/mL, Vector Labs, Burlingame, CA, USA) for one hour at room temperature. Following another TBS rinse, the sections underwent a last hour-long incubation with the avidin–biotin complex (ABC, 1:800, Vectastain Kit Elite, PK-6100, Vector Labs, Burlingame, CA, USA). After a final rinse, staining was shown through a reaction with a diaminobenzidine (DAB, Sigma-Aldrich, Burlington, MA, USA) solution with 0.01% hydrogen peroxide; this gives a brown stain where it reacts with the avidin–biotin complex. A positive control of this last reaction was accomplished by combining the remaining ABC with the DAB solution.

After a slight acidification with a 5% acetic acid solution, all slides were counterstained with a 2% cresyl-violet solution for 2 min, put through an ascending alcohol scale for dehydration, cleared in xylene, and mounted with Entellan new.

### 2.4. Image Acquisition and Analyses

All slides were systematically scanned using a semi-automated digital microscope (D-Sight, Menarini Diagnostics, Florence, Italy) at a 40 times magnification at the plane with the greatest clarity.

#### 2.4.1. Optic Nerve Cross-Sections

**Figure 1:**
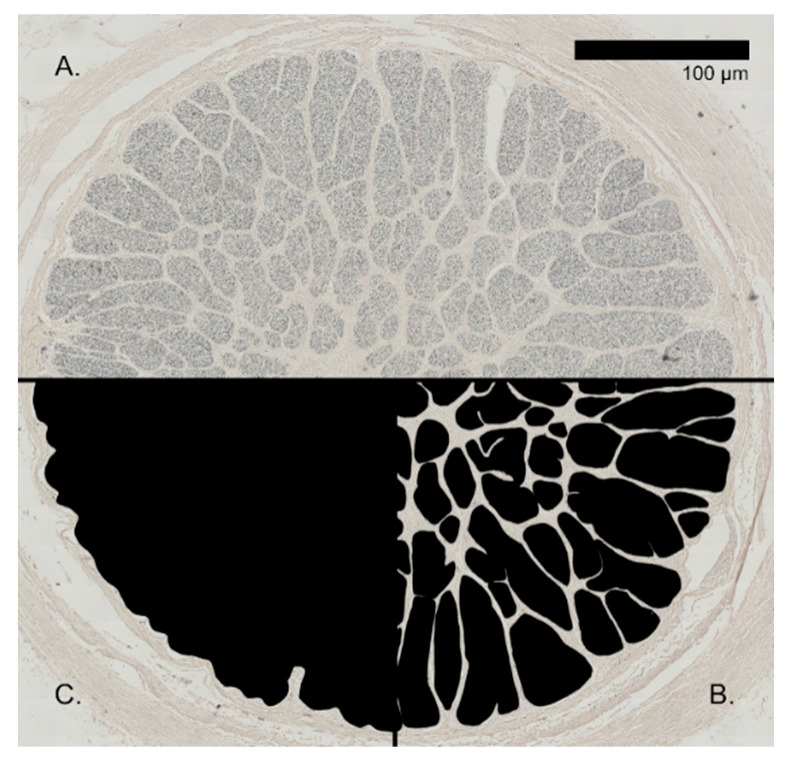
Illustration of the analysis of Weil-stained optic nerve sections. (**A**) Raw scan of *G. griseus*, (**B**) fascicles traced for measurement, (**C**) whole nerve (without nerve sheath) traced for measurement.

The optic nerve cross-section analysis included the manual tracing of the nerve bundles with glial cells and vasculature included and the entire nerve (without nerve sheath) through the GNU Image Manipulation Program (GIMP, version 2.10.30) as illustrated by Figure 1. These manual traces were then measured through ImageJ (version 1.53 k); with those measurements, the ratio of fascicular tissue to the total surface could be calculated as well as the number of nerve bundles. An estimate of axon count and density was not possible because of tissue deterioration.

#### 2.4.2. Retinal Cross-Sections

The retinal cross-sections treated with cresyl-violet were imported into the ImageJ software (version 1.53 k) after dividing the whole section into manageable pieces using GIMP. Retinal thickness and that of its constituting layers, as well as the number of ganglion cells, were measured in increments of 1000 µm across the retina. The ganglion cells were identified based on generally accepted criteria: position in the ganglion cell layer, a wide ring of cytoplasm with pronounced Nissl substance, and the clear presence of the nucleus with a distinct nucleolus [19,22]. All ganglion cells that were well focused were then separately exported for further analysis.

A Cellprofiler (version 4.2.1) pipeline was designed to extract quantitative measurements from those exports. The “identify primary objects” functionality was first implemented whereafter each predicted selection was manually tweaked where necessary and 230 shape and texture descriptors were derived. 

Data analysis was carried out in R studio (version 1.3.1093), and the resulting graphs were cleaned up in Inkscape (version 1.1.2). The linear density of the RGCs was compared between species using a Kruskal–Wallis test combined with a Dunn post hoc test, since their distribution was non-normal and variation was non-homogenous. The correlation between the linear density and the total retinal thickness was tested using Kendall’s rank test. 

A PERMANOVA test was applied to the measurements collected from Cellprofiler, and the “adonis2” function was used from the “vegan” package in R. A post hoc analysis using the “pairwise.adonis” function was used to ascertain between which species possible differences occurred, using a Bonferroni correction to produce an adjusted *p*-value. On top, a principal component analysis was performed on all ganglion cell measurements to give an indication about which descriptors capture the most variation in the data.

The slides on which immunohistochemistry was applied were visually inspected to ascertain which retinal layers showed a positive signal. The total number of immuno-positive ganglion cells was counted to calculate their percentage compared to the overall count.

## 3. Results

All the examined retinal samples showed the typical layering as previously described (see introduction) and showed positive immunohistochemical signals in various magnitudes. Staining varied significantly between different samples for the optic nerve, but nerve fascicles were still discernible.

### 3.1. Optic Nerve

Staining quality of the optic nerve varied significantly between different samples with strong degradation of the tissue visible in *P. crassidens*, *Z. cavirostris*, and *H. amphibius*. Even so, nerve fascicles were deemed adequately visible to give an accurate surface estimation in all samples. While the most important results will be mentioned here, the complete results of the analysis can be seen in Table 4. The division into nerve bundles varied strongly between species with no complete separation in *S. coeruleoalba* to almost 400 fascicles in the bovine. The total area of the nerve that contained axons in *G. melas* was only 75.11%, but it increased to 96.18% in *S. coeruleoalba*.

### 3.2. Retina

**Figure 2:**
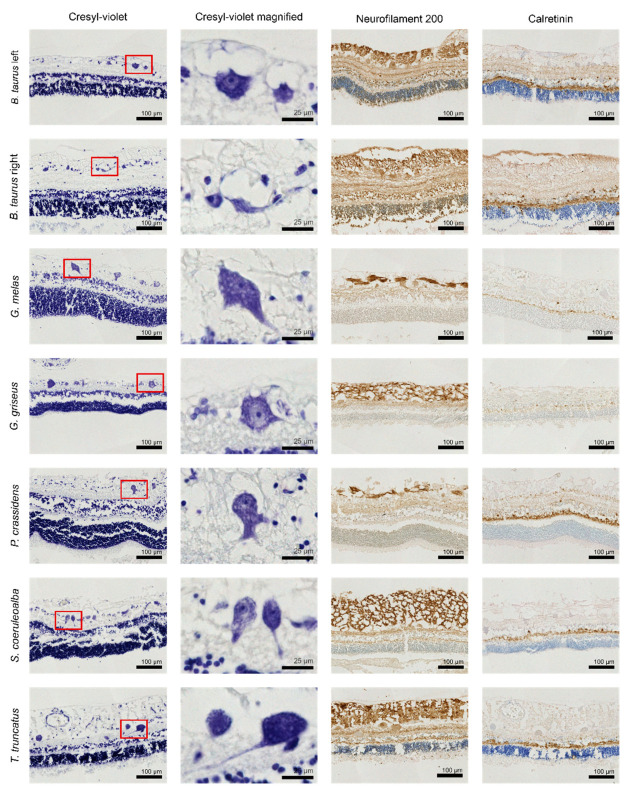
Scans of the retinal sections; the red rectangle represents the magnified view shown in column 2.

Even though the typical layered structure of the retina was visible, the processing protocol and sampling conditions did lower the quality of the tissue, which caused the separation of layers in certain samples and damaged small to moderate stretches of the cross-sections (see Figure 2). A total of 1826 ganglion cells were found, of which 737 (40%) were used for further processing.

#### 3.2.1. Retinal Thickness and Ganglion Cell Linear Density

The average value for the total retinal thickness, the mean linear density of RGCs per 1 mm fragment, the range of those values, and the total amount of RGCs per species can be found in Table 5. Differences in total amount are rather the result of missing segments of the retina within the sections than a real intra-specific correlation. A percentage of the ganglion cells were found within the inner plexiform layer or between the interneurons in the inner nuclear layer, reaching a notable 6.25% in *T. truncatus*.

**Figure 3:**
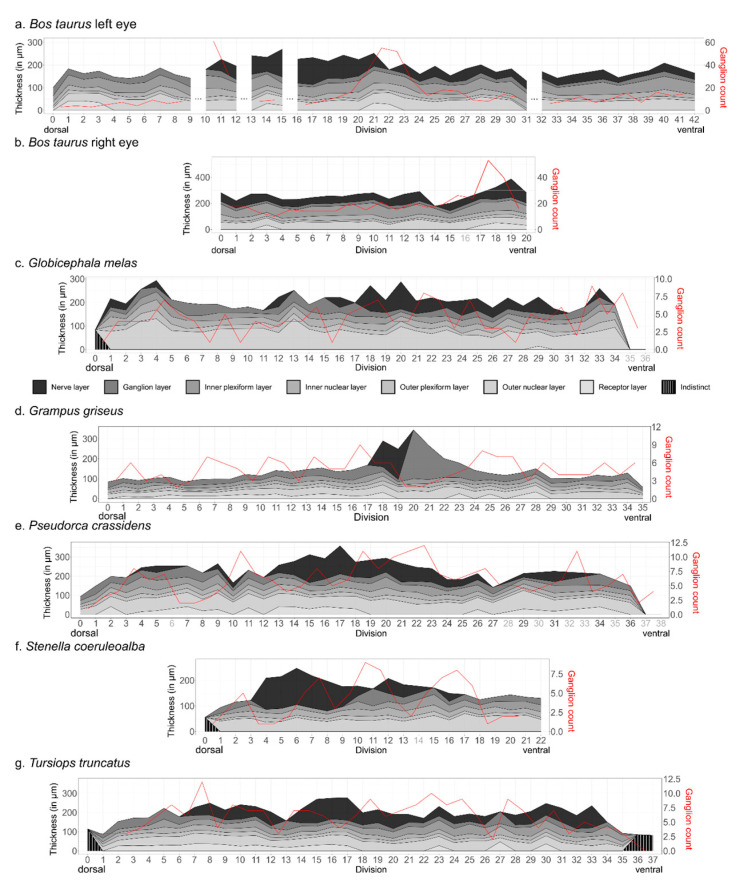
Figure representing the evolution of retinal ganglion cells (in red) and thickness across the eye. The number of divisions that were unsuitable for thickness measurements are indicated by a lighter grey colour; towards the peripheral sides of the eye, the layering was unclear, and the thickness measurements there are classed as indistinct.

A visual representation of the thickness of each separate layer and their evolution across the retina is given by Figure 3. The red line shows the progression of linear density of the ganglion cells. In some areas, the uppermost layer of the retina containing the axons of the ganglion cells was very distinct, showed a loose structure, and reached thicknesses that accounted for more than half the total thickness of the segment. Closer to the peripheral end of the retina, the layers were often indistinguishable; these are represented by the “indistinct” values on the graph. The divisions with light grey numbering represent parts where quantitative measurements were not possible. The thickness values there are local averages of the surrounding measurements.

A considerable variation in thickness can be seen between species and within species. This high variability within the samples could also clearly be seen visually. The mean thickness was highest in *B. taurus* (right eye) with a value of 263.8 µm; the highest value was found in this species as well at 387.4 µm of thickness. 

**Figure 4:**
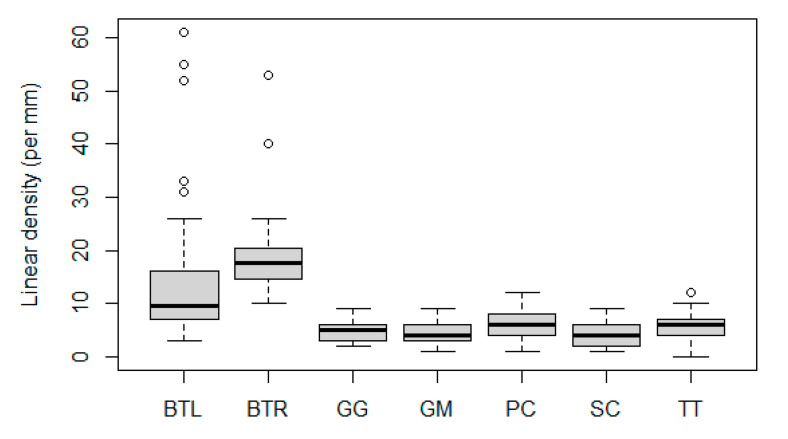
Boxplot of linear density per mm per species. BTL: *B. taurus* left eye, BTR: *B. taurus* right eye, GG: *G. griseus*, GM: *G. melas*, PC: *P. crassidens*, SC: *S. coeruleoalba*, TT: *T. truncatus*. Values outside of 1.5× the interquartile range are marked by points and classed as outliers.

The linear densities of the ganglion cells are visualised in Figure 4. The clear distinction between the distribution in values between the bovine and odontocete species was confirmed with the statistical analysis. The Kruskal–Wallis test indicated significant differences (*p*-value: 0), which the Dunn test confirmed were between both bovine samples and the cetacean samples. Within cetaceans, no significant differences were detected.

The peaks in the *B. taurus* samples represent a linear density of 61 and 53 RGCs per mm for the left and right eye, respectively. These point to the presence of high-density areas, corresponding to the visual streak, as described in the literature. The lowest density was found in *T. truncatus* with 0 cells per mm^2^.

The linear density was slightly but significantly correlated with the total retinal thickness with a tau value of 0.20 (*p*-value: 3.8 × 10^−5^). When this was investigated per species, no correlation was found for most species except for in *T. truncatus*, where the tau value was 0.31 with a *p*-value of 0.01.

#### 3.2.2. Retinal Ganglion Cell Measurements

**Figure 5:**
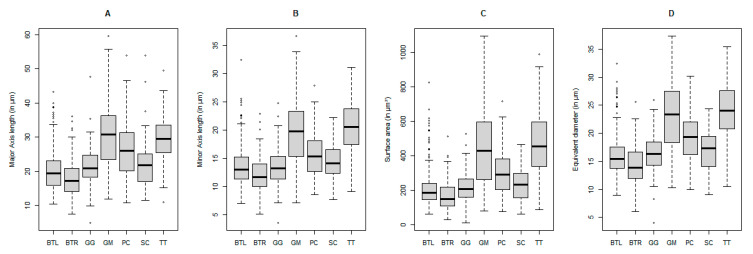
Distribution of major axis length (**A**), minor axis length (**B**), surface area (**C**), and equivalent diameter (**D**) of retinal ganglion cells in different species. BTL: *B. taurus* left eye, BTR: *B. taurus* right eye, GG: *G. griseus*, GM: *G. melas*, PC: *P. crassidens*, SC: *S. coeruleoalba*, TT: *T. truncatus*. Values outside of 1.5× the interquartile range are marked by dots and classed as outliers.

The variation and distribution in ganglion major axis length, minor axis length, cell surface area, and equivalent diameter per species are shown in Figure 5. Even though the range of values seem to overlap, a Kruskal–Wallis test did reveal that there are significant differences for these variables between certain species.

The cell with the biggest axis was found in *G. melas* at 59.5 µm, and even though the smallest RGC was found in *G. griseus* with a surface area of 12.8 µm^2^, the average value of each of those four descriptors was lowest in both bovine samples.

When all the descriptors were considered together for the PERMANOVA analysis, these differences were again confirmed with a *p*-value of 0.001, and it showed that 12.1% of the variation in retinal ganglion cell measurements could be attributed to species differences. The post hoc analysis showed no significant differences between the cells of *B. taurus* and *G. griseus* on one side and *G. melas*, *P. crassidens*, and *T. truncatus* on the other side based on the descriptors used in this study. Between those groups, however, the *p*-value was 0.021, and they were thus significantly different; the cells measured from *S. coeruleoalba* did not show any differentiation from the others.

**Figure 6:**
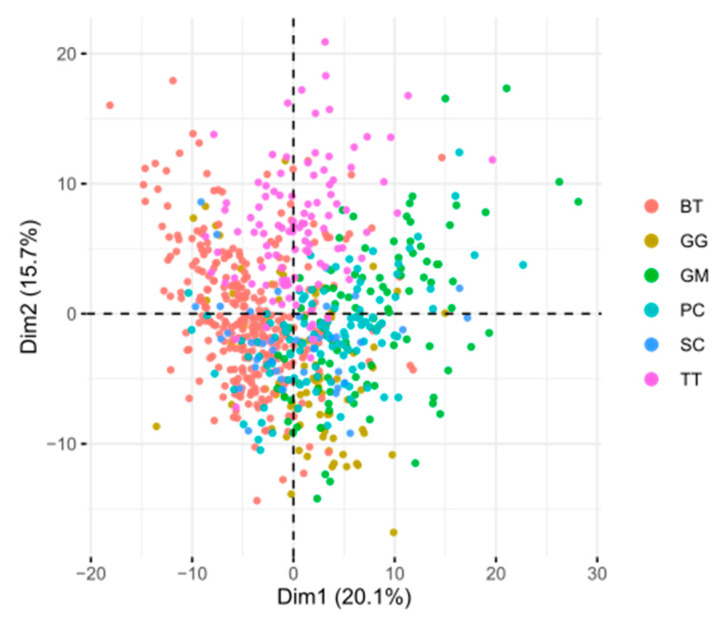
PCA biplot of all measurements across species. BTL: *B. taurus* left eye, BTR: *B. taurus* right eye, GG: *G. griseus*, GM: *G. melas*, PC: *P. crassidens*, SC: *S. coeruleoalba*, TT: *T. truncatus*.

The PCA analysis revealed that the variation in the data is difficult to compress into a lower number of dimensions, needing 16 principal components to capture 70% of the total variation. Each separate descriptor only made up a small percentage of each dimension. For PC1, the biggest contributor was entropy at 1.74%; this variable represents the complexity of the image (Figure 6).

### 3.3. Immunohistochemistry

All retinal cross-sections showed positive signals with differing intensities following the immunohistochemical procedures. While neurofilament 200 showed intense staining, calretinin antibodies produced a more muted result (Figure 2). In Table 5, the ratio of immunopositive ganglion cells to their total amount per antigen is shown.

#### 3.3.1. Neurofilament 200

Neurofilament 200 showed the strongest staining in the layer above the ganglion cell layer where the axons and arteries run. Most to all of the retinal layers showed some positive signal, mainly varying in whether or not the photoreceptors were stained. The percentage of immunopositive ganglion cells ranged from 31.42% in *P. crassidens* to 79.81% in *T. truncatus*.

#### 3.3.2. Calretinin

Calretinin showed the highest staining levels in the outer plexiform layer and within the inner nuclear layer primarily close to the edges and lighter signals in the inner plexiform layer. In only certain species did the ganglion cells show any staining: in *B. taurus* reaching 11.16% of the total ganglion cell count and in *T. truncatus* reaching 9.13%.

## 4. Discussion

### 4.1. Optic Nerve

The optic nerve structure showed distinctive configurations for each species with the ratio between fascicular and surrounding tissue and the division into fascicles showing significant variation. Cozzi et al. (2016) [17] state that the contribution of metabolically active tissue to the total optic nerve surface is higher in delphinids, reaching more than 50% of the total space. Even though we have obtained very low percentages of supportive tissue (only ~4% in *Stenella coeruleoalba* and 6.4% in *T. truncatus*), this ratio exceeds the expected mammalian range of 12–20% in *Globicephala melas*, *grampus griseus*, and *Pseudorca crassidens*. In *Z. cavirostris*, it does fall within that typical range with ~15%. Mazzatenta et al. (2001) [27] found a similar-looking nerve section for *S. coeruleoalba,* but the division in their study seems to be complete, while in our sample, strands of connective tissue did not fully separate segments. This could have resulted from the sample location behind the eye, as fascicles tend to split and merge along the nerve [5]. 

Samples from *G. griseus* and *G. melas* both came from newborn animals and display very similar values of the ratio of fascicular to total surface, which suggest that the age-related effect might have obscured any species-specific pattern. Effects of aging on the optic nerve include increased myelination and widening of the septa between fascicles in terrestrial mammals [36,37].

### 4.2. Retina

Supin, Popov and Mass (2001) [10] report a retinal thickness range of 370 to 425 µm for cetaceans and 110 to 220 µm for the bovine. While our results for the bovine are reasonably correct albeit quite high for the left eye (mean thickness 183.5 µm), the measurement for the right eye exceeds these values with an average value of 264.3 µm. The difference between both eyes could be explained by the loss of quite a large part of the retina of the right eye during processing, leaving out a considerable part of the peripheral retina. It also had a peak of 387.4 µm, skewing the average to a higher value. For the odontocetes, the average values reported here are much lower than expected, ranging from 140.2 to 228.7 µm. A partial explanation of that deficit could be in the detachment of the photoreceptor outer segments from the retina after processing. However, those segments were measured separately where possible and added to the thickness estimates, so the separation should not have an influence on the range of retinal thickness. We also might have simply missed the areas of highest thickness, since we only analysed one dorsoventral strip of the retina and thus did not account for any naso-temporal gradient.

From Figure 2, we can see that the outer nuclear layer, containing the cell bodies of the photoreceptors, occupies a larger proportion of total thickness in *G. melas* and *P. crassidens* than in the other species. Peichl (2005) [16] states that species which spend more time in low-light environments generally have higher photoreceptor densities and thus also a thicker outer nuclear layer. Both *G. melas* and *P. crassidens* forage for cephalopods at big depths, which gives a possible explanation [38,39]. However, *G. griseus* also targets that same food group and forages at great depths, and the thick outer nuclear layer is not apparent there [40]. Age could also be a factor in the variation in retinal thickness. Occelli et al. (2020) [41] reported an initial thinning of the retina over the first weeks of life in the dog, after which it stabilised. The samples from *G. melas* and *P. crassidens* came from newborn animals and showed the highest mean thickness of the odontocetes, 204.9 µm and 228.7 µm, respectively. However, Mass and Supin (2021) [42] reported an increase in retinal thickness from birth to adulthood in the common bottlenose dolphin. Future studies into these developmental patterns within different species could certainly prove interesting.

The difference in retinal ganglion cell density values between the bovine and cetacean samples agrees well with previous observations, showing a much reduced density in odontocetes [17]. A range of 4300 to 6500 cells per mm^2^ was found for the bovine by Hebel and Holländer (1979) [24]. Unfortunately, reference values are only known for three of the odontocete species included here: *G. melas* (241–294 cells/mm^2^ [22]), *P. crassidens* (475 cells/mm^2^ [26]), and *T. truncatus* (460–826 cells/mm^2^ [19,30]). Since some clustering was visible throughout the retina with small ganglion cell groups switched up with empty stretches, a simple conversion from linear density to total average density would be inaccurate. This prohibits the comparison of the densities found in this study to other studies.

Our study proved that there are significant interspecific differences based on the shape and texture descriptors considered here. However, species only explained about 12% of the variation seen within the retinal ganglion cells. No evidence was found for the existence of clearly delineated morphotypes of ganglion cells. However, the two types described by Dral (1975) [30] could also be seen: the first one having a round to oval outline, pale staining nucleus with fine tigroid cytoplasm and the other having a much darker tigroid, angular outline, and darkly stained nucleus. It is possible that the descriptors we used for texture did not sufficiently pick up on these characteristics, or it might not be enough of a dividing quality, since many intermediate appearances were also found.

The expected pattern of the higher size distribution of ganglion cells in the cetacean samples compared to the bovine samples can be seen from our data, although the differences in equivalent diameter were not always significant for each species separately. Previous studies reported values between 9 and 60 µm for the common bottlenose dolphin with most cells having a diameter between 20 and 35 µm [19]. This is confirmed by our results, with a range of 10.5–35.5 µm in equivalent diameter with an average of 24.1 µm. The ‘giant’ ganglion cells are also characteristic of the cetacean retina, which reach diameters of up to 75 µm and more [22,43]. Cells of those size could not be found in our samples, but each cetacean did have cells with a major axis higher than 45 µm, reaching up to 59.5 in *G. melas*, already exceeding the limit of 15–35 µm used for terrestrial mammals to denote giant cells [10]. The absence of these bigger cells could have resulted from the long periods spent in fixative for some of the samples, ranging from several days for the bovine to multiple years in other species. Finnie et al. (2021) [44] report strong influences of the neutral-buffered formalin on the retina. Especially relevant here are the shrinkage of the cytoplasm of the ganglion cells and pyknosis leading to eventual disappearance. This process is also coupled with the formation of clear, vacuolated spaces resulting in a substantial expansion of the ganglion cell layer. The latter phenomenon could be clearly seen in our samples (see Figure 2), thus indicating that the fixation had a significant overall effect. 

### 4.3. Immunohistochemistry

Neurofilament 200 showed heavy staining, making it challenging to concern legitimate positive signals from background staining. All layers of all species showed staining, but it was most intense within the innermost layer where the axons run. Neurofilament 200 is said to selectively label large ganglion cells [45], but in our samples, the diameter of the positive cells covered the whole reported range. 

Calretinin showed consistent staining of the outer plexiform layers, the interneurons close to the edges of the inner nuclear layer, and a portion of retinal ganglion cells in *T. truncatus* and *B. taurus*. Although the physiological role of calretinin in the cell remains unclear, it has previously been reported to differentially stain types and subtypes of retinal cells [46]. While the neuronal cells close to the outer nuclear layer can confidently be identified as horizontal cells here, they do not stain with calretinin in both mouse and human [47,48]. On the other side of the inner nuclear layer, the stained cells are most-likely amacrine cells; these regulate the signals the bipolar cells transfer to the ganglion cells. Calretinin consistently stains amacrine cells in most examined species, including in mice, hamsters, and humans [49]. Calretinin-positive ganglion cells are present as different morphological subtypes in numerous species; in the mouse, about 85% of all ganglion cells showed a positive signal [44]. In Mysticeti, a similar pattern could be seen with the intense staining of horizontal and amacrine cells; however, no positive signal was found in the ganglion cell layer [50]. 

The differences in expression of these immunogens show promise for further chemical variation within the odontocete retina and warrant further study.

### 4.4. Limits of the Present Study

Since most of the samples studied here come from wild animals and are quite rare, we could only obtain one sample per odontocete species. This limits our ability to distinguish individual variation from interspecies differences. Due to practical constraints, only one section per species was investigated, which complicates the interpretation of the results, since variation within one section might not accurately reflect all variation within a singular species. For example, Buttery et al. (1991) [51] found that retinal thickness does change across a naso-temporal gradient as well as the ventrodorsal gradient investigated here.

Differences in both the retina and optic nerve cannot be attributed solely to species differences as we can see from the variation within bovine samples. However, we can still see that the sections from the bovine are more similar to each other than to the other species. The samples also fluctuated in age and sex of the specimen, but species with equal values for those characteristics, e.g., *T. truncatus* and *G. griseus*, still showed significant differences.

Furthermore, tissue quality fluctuated significantly between the different samples due to the differing post-mortem intervals; this is the time between death and fixation. It is difficult to estimate this accurately for stranded animals, so only samples with a decomposition condition category below three were used. Advanced tissue degradation and prolonged fixation can diminish immunoreactivity [52] and alter certain tissue characteristics, e.g., by causing tissue swelling and homogenisation [42].

## 5. Conclusions

In conclusion, we were able to show differences in varying aspects of the neuronal elements of the ocular system between different odontocete species and with our reference species. Some of our observations align well with what is known in the literature, while others show some deviation, like retinal thickness. Anatomical characteristics of the optic nerve appear mostly species specific, while ganglion cell density was higher in the terrestrial bovine than in the cetaceans. The retinal ganglion cell size did show variation between species, but this was not significantly different between all species (for example, the toothed whale values overlapped with those of the bovine). Both neurofilament 200 and calretinin selectively stained portions of the retinal ganglion cell population, and this sets the stage for further immunohistochemical characterisation of the odontocete retina.

## Figures and Tables

**Table 1:** Reference values for retinal ganglion cell and optic nerve attributes for the species included in present study. RGC: retinal ganglion cell, ON: optic nerve.

Species	Peak RGC Density (Cells/mm^2^)	RGC Diameter Range (in µm)	Mean Fibre Density of ON (Axons/mm^2^)	Axon Diameter Range (in µm)	Data from
*Bos taurus*	4300–6300	3.5–35			[24]
*Globicephala melas*	241–294	10–75			[22]
*Grampus griseus*	No data available				
*Hippopotamus amphibius*	1800				[25]
*Pseudorca crassidens*	475				[26]
*Stenella coeruleoalba*			24,100	0.5–15.2	[27]
*Tursiops truncatus*	460–826	9–60	19,053–77,000	0.27–25.6	[19,28,29,30]
*Ziphius cavirostris*	No data available				

**Table 2:** Sample data with final use; R + O: retinal and optic nerve cross-sections, ON: optic nerve sections only, PMI: post-mortem interval, DCC: decomposition condition category.

Species	Common Name	Sex	Age	Cause of Death	Use	PMI/DCC
Artiodactyls						
*Bos taurus*	Bovine	Unknown	Adult	Commercial slaughter	R + O	4 h
*Hippopotamus amphibius*	Common hippopotamus	M	46	Cardiac arrest	ON	<24 h
Odontocetes						
*Globicephala melas*	Long-finned pilot whale	F	Newborn	Unknown	R + O	DCC 1
*Grampus griseus*	Risso’s dolphin	F	Adult	Septic shock	R + O	DCC 1
*Pseudorca crassidens*	False killer whale	F	Newborn	Unknown	R + O	DCC 2
*Stenella coeruleoalba*	Striped dolphin	M	Juvenile	Caudal fin lesion	R + O	DCC 1
*Tursiops truncatus*	Common bottlenose dolphin	F	Adult	Unknown	R + O	DCC 1
*Ziphius cavirostris*	Cuvier’s beaked whale	M	Juvenile	Unknown	ON	DCC 2

**Table 3:** Primary and secondary antibodies used in present work.

Antibody	Production Method	Product Details	Dilution
Anti-CR	Produced by immunisation of mice with recombinant human calretinin-22 k (identical with calretinin up to Arg178)	Swant, mouse monoclonal, Cat# 6B3, Lot n° 010,399, RRID: AB_10000320	1:500
Anti-N200	Produced by fusion of mouse myeloma cells and splenocytes from a mouse immunised with neurofilament 200 from porcine spinal cord	Sigma-Aldrich, mouse monoclonal, Cat# N5389, Lot n° 050M4779, RRID: AB_260781	1:500
Anti-mouse	Horse anti-mouse antibodies directed at both heavy and light chain	Vector Laboratories, Cat# BA-2000, RRID: AB_2313581	1:400

**Table 4:** Result of the optic nerve analysis.

Species	Total Nerve Surface Area (in mm^2^)	Total Fascicular Surface Area (in mm^2^)	Amount of Nerve Bundles	Percentage of Fascicular Surface to Total
*Bos taurus* (left eye)	3.973	3.138	329	78.98
*Bos taurus* (right eye)	3.796	3.059	393	80.58
*Hippopotamus amphibius*	2.174	1.777	92	81.74
*Globicephala melas*	1.591	1.195	54	75.11
*Grampus griseus*	3.241	2.586	167	79.79
*Pseudorca crassidens*	2.527	1.916	108	75.82
*Stenella coeruleoalba*	2.014	1.937	1	96.18
*Tursiops truncatus*	2.986	2.795	4	93.60
*Ziphius cavirostris*	3.450	2.959	49	85.77

**Table 5:** Result of the analysis of the retinal cross-sections. RGC: retinal ganglion cell, LD: linear density of RGCs, N200: neurofilament 200, CR: calretinin.

Species	Mean Thickness (in µm)	Thickness Range (in µm)	Total RGCs	Mean LD (per mm)	Range LD (per mm)	N200+ Ratio	CR+ Ratio
*Bos taurus* (left eye)	183.5	183.5–270.1	563	15	3–61	49.41	11.16
*Bos taurus* (right eye)	263.8	180.6–387.4	402	20	10–53	53.66	10.57
*Globicephala melas*	204.9	83.7–293.7	158	4	1–9	69.59	0
*Grampus griseus*	140.2	58.1–345.1	171	5	2–9	51.66	0
*Pseudorca crassidens*	228.7	93.7–358.3	231	6	1–12	31.42	0
*Stenella coeruleoalba*	160.2	55.7–247.8	87	4	1–9	65.69	0
*Tursiops truncatus*	192.5	81.3–277.7	103	6	0–12	79.81	9.13

## Data Availability

All data are available from the corresponding author on request.
